# Advances in CRISPR Plant Applications

**DOI:** 10.3390/ijms27094095

**Published:** 2026-05-03

**Authors:** Leo Jing, Devjyoti Roy, Melanie Kalischuk

**Affiliations:** Department of Plant Agriculture, University of Guelph, Guelph, ON N1G 2W1, Canada; leo.jing@queensu.ca (L.J.);

**Keywords:** immunity, RNA, gene editing, crop improvement

## Abstract

The ability to precisely edit genetic characteristics with a CRISPR (clustered regularly interspaced short palindromic repeats)/Cas (CRISPR-associated) immunity complex is a revolutionary advance in science. Originally discovered in bacteria as part of a natural defense mechanism against viruses, CRISPR/Cas provides a precise, efficient, and relatively simple method for editing genes in microbes, plants, animals, and humans. The process relies on the Cas protein, an enzyme that cleaves and unwinds DNA at targeted locations. This process is guided by RNA sequences complementary to the DNA or RNA sequence of interest, allowing for changes to the genome through innate non-homologous end joining (NHEJ) and homology-directed repair (HDR). The potential applications of CRISPR/Cas are immense and, in agriculture, is facilitating crop development with resistance to abiotic, biotic, and agronomic characteristics that improve yield, quality, and food security. Gene editing also facilitates the relatively rapid modification of regulatory and complex pathways that enable studies to advance our understanding of gene function. This review provides an update of the fast-evolving CRISPR/Cas modification of important crops to address emerging global population, as well as environmental and climate challenges.

## 1. Introduction

Traditional plant breeding and transgenic/cisgenic genetically modified plants/organisms (GMOs) have improved crop production, but their capabilities have been restricted by relatively long timelines, lack of precision, and acceptance limitations. Precise genetic engineering methods have emerged, but application of zinc finger nucleases (ZFNs) and Tal effector nucleases (TALENs) has not been widely applied because of the complexity [1,2]. A relatively simple method for site-specific gene editing was later discovered in prokaryote adaptive immunity, involving clustered regularly interspaced short palindromic repeats (CRISPR) and CRISPR-associated (Cas) nuclease [3]. Remarkably, a non-coding single guide RNA (sgRNA) can target nucleic acid and direct a Cas endonuclease to cleave it with specificity, after which the genomic site is repaired by innate cellular repair mechanisms.

In 1987, scientists discovered an unusual, interrupted repeat of sequences in *Escherichia coli* [4]. Subsequently, the terms Short Regularly Spaced Repeats (SRSR) and CRISPR were introduced to describe the increasing reports of similar sequences [5,6]. A key advance in our understanding of CRISPR was the observation that the clustered repeats were associated with CRISPR-associated (Cas) proteins possessing helicase and nuclease motifs [6]. Several breakthroughs indicated spacers matched viral sequences indicating an adaptive immunity mechanism, identified the Cas9 sequence and adjacent Protospacer Adjacent Motif (PAM), and confirmed bacterial immunity against phage infection in prokaryotes with application in eukaryotes [7,8,9,10,11,12,13,14]. Further characterization identified essential non-coding CRISPR RNA (crRNA) as the guiding sequence and trans-activating CRISPR (tracrRNA) that complexes with crRNA to secure Cas protein and target DNA for cleavage. In *Streptococcus pyogenes* the CRISPR Cas9 complex includes a trans-activating CRISPR RNA combined into a single guide RNA (sgRNA) and the crRNA may be synthesized to target key sequences for editing [3]. A simplified engineered two-part CRISPR-Cas9 in vitro system was developed to facilitate gene editing of genomes in human and animal cells that allows precise changes across a wide range of organisms. This review focuses on the rapidly increasing number of plant traits being edited with CRISPR-Cas platforms to produce stress-resilient and high value-added crops.

## 2. Crop Applications

**Figure 1:**
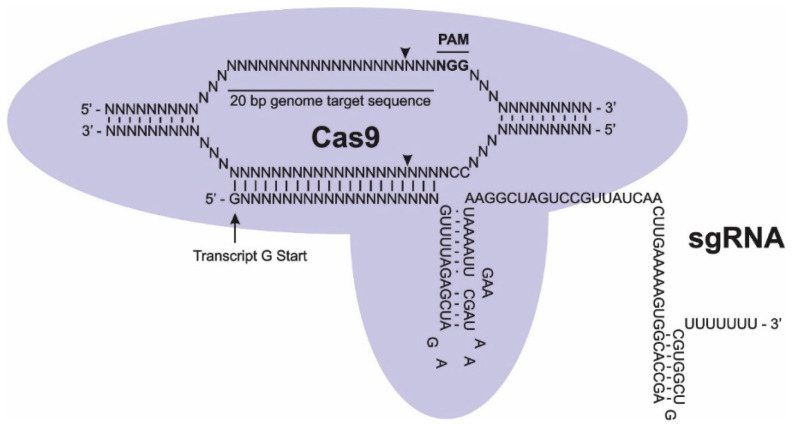
A schematic illustration of the clustered regularly interspaced palindromic repeats (CRISPR) single guide RNA (sgRNA)and Cas endonuclease. The sgRNA consists of the N_20_ CRISPR RNA (crRNA) guide sequence and the trans-activating RNA (tracrRNA) that acts as a scaffold for Cas recognition. Restriction (triangles) of the targeted DNA occurs 3 nucleotides 5′ to NGG Protospacer Adjacent Motif (PAM) sequence and sgRNA transcript starting with 5′G transcription initiation nucleotide using the U6 promoter.

Initial plant CRISPR-Cas genome editing exploited DNA double-strand breaks (DSBs) created by the Cas enzyme, guided by a sgRNA, that cleaves the DNA at the specific crRNA target sequence—3 nucleotides 5′ of the PAM site [15,16,17]. The sgRNA specificity is conferred by its 5′ sequence and is usually 20 nucleotides in length N_20_NGG (Figure 1). Plant RNA polymerase III promoters such as U6 are required to ensure a defined transcription start nucleotide of the sgRNA. Delivery of the sgRNA may involve protoplasts, callus, or axillary meristems either through direct delivery with Cas sequence or via transformation using *Agrobacterium tumefaciens* [18]. Repairs may be precise using homology-directed repair (HDR) or unpredictable by non-homologous end joining (NHEJ), producing mutations, insertions, or deletions. Introduction of missense, nonsense, or frameshift sequence usually produces knockout mutations or functional changes that can alter trait characteristics. In plants that are polyploid, the mutation may be epistatic and masked if recessive, but crops have been successfully edited and selected through screening to deliver the desired phenotype [15,16,17,18]. An insertion is also possible through HDR by the addition of an epigenetic naked DNA or chromatin template, that is flanked by homologous sequence to guide DSBs for error-free repair, with chromatin donor matrices delivering higher editing efficiencies. Edits using a Cas9 substitute such as Cas12a (Cpf1), a cytosine base editor that does not require a double-strand DNA cleavage, or Cas13 that recognizes single-stranded RNA (ssRNA), increases the flexibility and precision of the sequence modifications [19,20].

## 3. RNA Adaptive Immunity in Plants

Plant genetic research has been transformed by remarkable innate molecular mechanisms, including CRISPR gene editing and RNA silencing [3,21]. Both technologies exploit RNA-activated immunity and allow modulation of gene expression, but they operate through distinct mechanisms and have different applications in plant biology and biotechnology. Understanding their similarities and differences is essential for selecting the appropriate method for specific research or crop improvement objectives. The CRISPR-Cas system enables precise and stable modifications of plant genomes using sgRNA to direct a Cas nuclease to a specific DNA sequence, producing a DSB. The cleaved nucleic acid is repaired by either NHEJ, that often introduces small insertions or deletions, or HDR, allowing for larger precise gene modifications [3]. RNA silencing, also known as RNA interference (RNAi), is a post-transcriptional gene regulation mechanism that generates small RNA molecules (sRNA), such as small interfering RNAs (siRNAs) or microRNAs (miRNAs). These molecules bind to virus double-stranded RNA (dsRNA) or complementary messenger RNA (mRNA) leading to degradation or inhibition of translation [21]. This process does not alter the genomic DNA but rather downregulates replication and gene expression, either transiently or stably. Applications of antisense RNA have grown rapidly since the initial reports of its ability to alter phenotype and obtain high levels of disease resistance [22,23]

Mechanisms of adaptive RNA immunity differ in that CRISPR-Cas gene editing is a DNA-level modification via nuclease activity and silencing operates at the RNA-level through small RNA-mediated degradation by the RNA-induced silencing complex (RISC) [21]. Whereas CRISPR-Cas typically results in permanent genomic changes, RNAi may be transient or semi-stable depending on the delivery method. Both CRISPR-Cas and RNAi are highly precise, but off-target mutations or unexpected expression downregulation may occur. Regulatory agency evaluation is similar for RNA immunity products, but consumer acceptance is often influenced by the delivery method and novelty of the plant trait combination. RNAi is often effective for transient applications of exogenously applied double-stranded RNA (dsRNA) to target genes, especially when combined with nanoparticle protection and optimized delivery [24,25]. While RNAi is ideal for temporary gene silencing and functional genomics studies, CRISPR is often applied in genome editing, gene therapy, and studying gene function [3]. CRISPR-Cas is particularly well-suited for creating knockouts, correcting alleles, and engineering cis-regulatory elements, offering precision breeding opportunities without necessarily introducing foreign DNA. In summary, RNAi and CRISPR are powerful adaptive immunity tools for gene manipulation, but their mechanisms, targets, and applications differ significantly.

## 4. CRISPR-Cas Characterization

**Figure 2:**
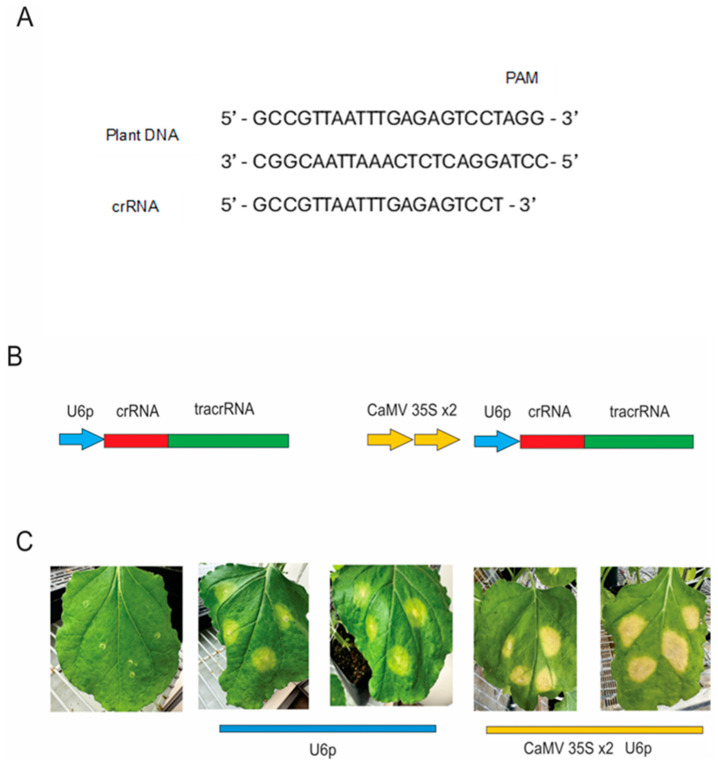
Application CRISPR-Cas to facilitate vector development and determine editing efficiency. (**A**) Illustration of crRNA sequence for phytoene desaturase (PDS) in *Nicotiana benthamiana* in an sgRNA Cas complex [18,26]. (**B**) Schematic diagram of DNA constructs used for expression and characterization of impact with the PDS visual reporter crRNA and tracrRNA [26]. (**C**) Agroinfiltration of *N. benthamiana* leaves 3 weeks post-infiltration showing impact of the duplicated CaMV 35S promoter–enhancer as previously described [18,26,30].

Agroinfiltration is considered a transient sequence delivery procedure that utilizes *Agrobacterium tumefaciens* strains, such as GV3101 or EHA105, to deliver foreign DNA into plant tissues [26]. Frequently used in gene expression studies, agroinfiltration has been widely adapted for functional genomics and genome editing research, notably with CRSPR Cas systems. This simple, cost-effective, rapid, and efficient procedure has made agroinfiltration an invaluable method for characterization of CRISPR targets and constructs (Figure 2). Screening of vector and construct efficiency in generating gene edits may be determined by reporter genes such as luciferase, fluorescent proteins, or phytoene desaturase (PDS) [27,28,29]. Delivery into protoplasts or leaves and axillary meristems through agroinfiltration facilitates the relatively rapid determination and optimization of the expression vectors and regulatory elements. For example, expression of sgRNA and Cas may be elevated with the use of an enhancer such as the duplicated CaMV 35S promoter [30]. A combination of the enhancer and a promoter with a defined transcription start nucleotide to produce sgRNA can increase the efficiency of gene editing (Figure 2). Observation of edited tissue culture or soil-propagated plants occurs within weeks and allows for relatively fast widescale screening of sgRNA vectors and production of gene-edited tissues.

## 5. CRISPR-Cas Delivery

**Figure 3:**
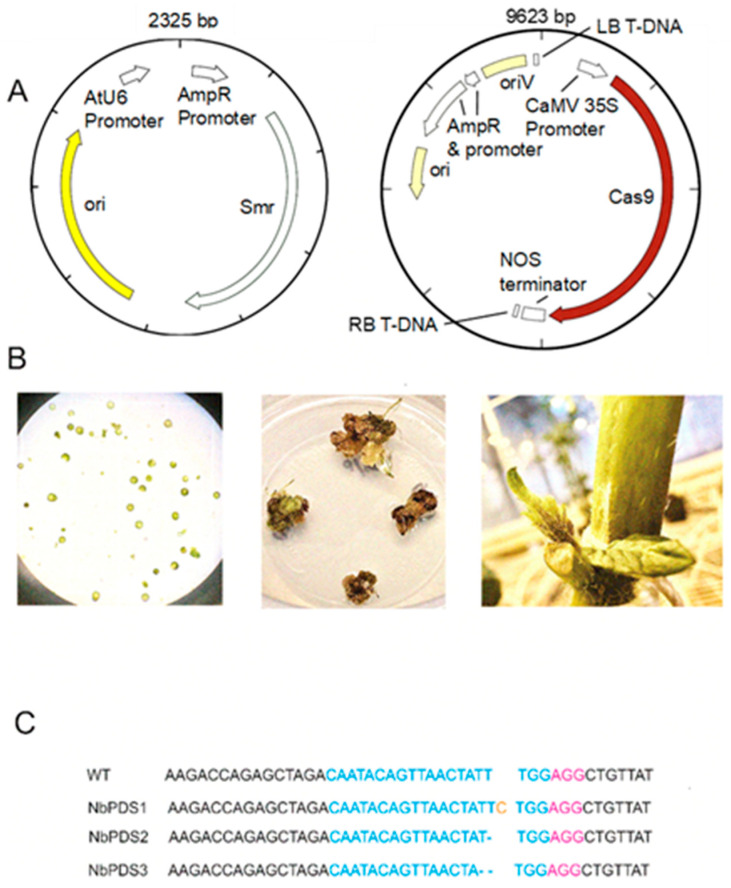
Introduction of CRISPR-Cas into plants for sequence editing. (**A**) Binary vectors for *Agrobacterium tumefaciens*, simultaneously expressing sgRNA with desired crRNA (blue) target sequence and PAM site (pink) [33,34]. (**B**) Methods for transitional transformation include protoplasts (**left**), wounded tissue and callus formation (**middle**), and axillary meristems (**right**) producing adventitious shoots (**right**) [18]. (**C**) Sequence of phytoene desaturase (PDS) editing detected following agroinfiltration of *Nicotiana benthamiana* leaves 3 weeks post-infiltration identifies a nucleotide insertion (gold) and deletions (dashes) as described by previous studies [18,26].

Several procedures are available for stable plant genome knockout or gain of function editing (Figure 3). Protoplasts allow direct delivery of sgRNA and Cas but are relatively slow, requiring the regrowth of cell walls and use of phytohormones that can cause undesirable somaclonal variation and off-target phenotypes [31]. Production of callus from wounded tissues with *A. tumefaciens* delivery of sgRNA and Cas reduces exposure to phytohormones and shortens plant production timelines. In contrast, axillary meristems can produce gene-edited adventitious shoots within weeks in culture-free propagation with minimal need of phytohormones and developmental regulators, but they often produce chimeric tissues [18,32]. Procedures and strategies for introducing CRISPR-Cas for plant editing, including Agrobacterium-mediated transformation, biolistics, transfection, nanoparticle-mediated delivery, and injection have been developed and well-characterized for protoplasts, calli, and explants to maximize efficiency for each plant trait modification [18,26,33,34].

## 6. Abiotic Stress Resilience: Single Genes to Network Modulation

Abiotic stresses, including drought, salinity, extreme temperatures, and oxidative stress, remain major constraints on crop productivity under changing climates and intensifying production pressures. Although traditional breeding has improved stress tolerance, progress is often slow because these traits are typically quantitative, polygenic, and strongly influenced by environmental conditions. Transgenic strategies have introduced beneficial alleles, but regulatory barriers and public concerns have limited widespread adoption. The emergence of CRISPR–Cas genome editing has shifted this paradigm by enabling precise modification of endogenous genes and regulatory elements, allowing targeted changes in stress-response networks without necessarily introducing foreign DNA. One of the most significant advances is the capacity to modulate hormone signaling pathways that integrate environmental sensing with growth regulation (Table 1). For example, editing ABA-related regulators such as *AREB1* can enhance drought responsiveness by fine-tuning stomatal closure and water-use efficiency, while modifications to *OST2* can alter guard cell dynamics [35,36]. Importantly, these examples illustrate a broader shift in engineering strategy; rather than constitutive overexpression of stress-response genes, that often imposes growth penalties, CRISPR enables adjustment of signaling sensitivity, thereby balancing stress tolerance with yield stability.

Applications targeting salinity and temperature tolerance further demonstrate how genome editing can refine key regulatory nodes in plant stress physiology. Editing ion transporters such as *ZmHKT2* improves sodium exclusion or compartmentalization, enhancing ionic homeostasis under saline conditions, while disruption of negative regulators such as *HyPRP1* in tomato increases survival under high salinity [56,60]. However, these interventions also highlight an important limitation; modification of ion transport or hormone signaling pathways can cause pleiotropic effects on development and nutrient balance. Consequently, emerging approaches emphasize multiplex editing and promoter engineering to control when and where target genes are expressed, rather than relying solely on gene knockouts.

A similar transition toward regulatory network engineering is evident in temperature stress responses. CRISPR-mediated manipulation of heat shock factors (HSFs), heat shock proteins (HSPs), and upstream regulators such as *SlAGL6*, *ZmTMS5*, and *SlMAPK3* enhances thermotolerance by stabilizing proteins and reproductive tissues during heat stress [68,70,72], while editing targets including *OsMYB*, *VInv*, and proline-rich proteins improves cold tolerance through increased membrane stability and osmolyte accumulation [74,77,85]. Herbicide resistance was similarly obtained by editing the acetolactate synthase *OsALS*, which is involved in amino acid synthesis [95], and the polyamine uptake transporters *OsPUT*, which improved paraquat resistance without yield loss [97]. Collectively, these studies underscore that CRISPR’s greatest potential lies not simply in gene disruption but in reshaping transcriptional and metabolic pathways that coordinate stress adaptation. Because abiotic tolerance is rarely governed by a single locus and often reflects quantitative trait loci (QTL) architecture, future progress will depend on multiplex editing of pathway components, integration with genomic selection, and the use of transcriptomic and epigenomic data to identify central regulatory hubs rather than peripheral stress markers.

## 7. Elevating Biotic Resilience: Immunity and Ecological Interactions

CRISPR-Cas genome editing, derived from a prokaryotic adaptive immune system, has emerged as a powerful platform for engineering plant responses to biotic stress. Its applications extend across resistance to pathogens, insect pests, and nematodes, as well as the modulation of beneficial plant–microbe interactions (Table 2). Although pesticides, crop rotation, and conventional breeding have historically mitigated biotic stress, these strategies often lack genetic precision, require lengthy breeding cycles, or raise environmental and health concerns. Moreover, resistance breeding is frequently constrained by available germplasm and linkage drag, limiting the speed and specificity with which desirable traits can be deployed. In contrast, CRISPR–Cas enables targeted editing of genes controlling immunity, defense signaling, and host–pathogen compatibility [3], thereby facilitating both incremental improvements and the creation of novel resistance phenotypes through precise modification of coding or regulatory sequences (Table 2).

A prominent strategy involves disruption of plant susceptibility *S* genes, which are host factors exploited by pathogens during infection. For example, knockout of *MLO* alleles across multiple crops confers durable resistance to powdery mildew [101,102,103], while CRISPR-mediated mutation of *OsSWEET* genes in rice blocks pathogen-induced activation of sugar transporters by *Xanthomonas oryzae*, enhancing resistance to bacterial blight without detectable developmental penalties [115]. These cases demonstrate how loss-of-function edits can generate broad-spectrum disease resistance while minimizing the growth–defense trade-offs often associated with constitutive activation of immune pathways. Similarly, CRISPR has been used to engineer resistance to plant viruses and viroids either by directly targeting viral genomes or by modifying host factors required for viral replication [123,124,125,126,127,128,129,130,131,132,133,134,135,136,137,138,139]. Although these strategies demonstrate considerable versatility, their long-term durability remains influenced by viral mutation rates and the potential emergence of escape variants. 

Beyond pathogens, CRISPR–Cas provides opportunities to strengthen endogenous defenses against herbivores and nematodes while also enabling the rational engineering of beneficial plant–microbe interactions. Unlike transgenic approaches that introduce exogenous toxins, genome editing can enhance native defense pathways, potentially improving regulatory acceptance and ecological compatibility. For instance, modification of cytokinin oxidase/dehydrogenase *OsCKX* genes in rice influences jasmonic acid-mediated insect defense signaling, increasing resistance to chewing and sucking pests [140], while knockout of *GmGUT* genes in soybean alters flavonoid biosynthesis and enhances resistance to chewing insects through relatively minor genomic changes [142]. Resistance to nematodes has likewise been achieved through editing of susceptibility genes such as *HPP04* in rice and *SNAP02* in soybean [143,144], reinforcing a broader concept that CRISPR-mediated resistance often arises from removing host compatibility factors or modulation of defense metabolism rather than introducing new resistance genes.

Increasing attention is also being directed toward engineering beneficial symbioses with organisms such as arbuscular mycorrhizal fungi and nitrogen-fixing bacteria. Because these associations rely on finely regulated signaling networks, precise genome edits can be particularly advantageous. For example, modification of genes in the common symbiosis signaling pathway, including *SYMRK*, *CCaMK*, and *CYCLOPS*, may enhance mycorrhizal colonization efficiency [146], while manipulation of the flavone biosynthetic pathway in rice promotes bacterial biofilm formation and improves biological nitrogen fixation, resulting in increased seed yield [147]. Collectively, these studies reflect a shift from purely resistance-based paradigms toward optimization of plant holobiont function. As the understanding of plant–pathogen–pest–microbe interactions expands, CRISPR–Cas will remain an essential tool for refining immune recognition, regulating defense networks, and improving crop resilience within next-generation crop improvement strategies [148,149,150].

## 8. Phenotype and Agronomic Performance Improvement

CRISPR-Cas9 genome editing has been successfully applied in a range of crop plants to enhance yield, improve quality traits, and boost agronomic performance (Table 3). These advances are increasingly important for meeting the demands of a growing global population while supporting food security and the development of more resilient and efficient agricultural systems. By enabling precise modification of genes controlling plant growth, metabolism, and environmental responses, CRISPR not only accelerates crop improvement but also expands the range of traits that can be modified beyond the constraints of traditional breeding.

Yield improvement efforts have largely focused on genes regulating plant architecture, reproductive development, and resource allocation. For example, CRISPR-mediated editing of the rice grain number gene *Gn1a*, which regulates cytokinin degradation, has generated loss-of-function alleles that increase cytokinin levels and enhance grain number and yield [165]. Similarly, editing of *PYL* genes involved in abscisic acid signaling has produced rice plants with optimized architecture and improved grain production [167]. In tomato, targeted modification of *SlIAA*, a regulator of parthenocarpy, and the polygalacturonase gene *PG* has resulted in seedless fruit and delayed softening, respectively, improving both productivity and postharvest shelf life [172,173]. These examples highlight how CRISPR enables direct manipulation of developmental pathways that were previously difficult to modify with precision.

Beyond yield, CRISPR-based potato varieties with modified granule-bound starch synthase *StGBSS* s show altered starch composition [175], while disruption of the inositol tetrakisphosphate kinase gene *BnITPK* in oilseed crops reduces phytic acid content, improving mineral bioavailability and nutritional quality [151]. In wheat, modification of *TaGW2*, a negative regulator of grain weight, increases grain size and mass, contributing to improved baking quality and market value [186]. Genome editing has also been used to improve crop performance under diverse environmental conditions. In soybean, editing of flowering-time genes *GmFT2a* and *GmFT5a* has enabled the development of varieties better adapted to different latitudes and seasonal conditions, supporting broader cultivation and improved yield stability [155]. Collectively, these studies illustrate CRISPR’s versatility in refining developmental, metabolic, and adaptive pathways to simultaneously enhance yield potential, nutritional quality, and environmental resilience. As plant genomic and transcriptomic resources continue to expand, the identification of new gene and regulatory targets will further increase the precision and scope of genome editing strategies, positioning CRISPR as a central tool for developing crop varieties capable of meeting evolving agronomic, industrial, and societal demands.

## 9. Future Applications of Gene Editing in Plants

**Figure 4:**
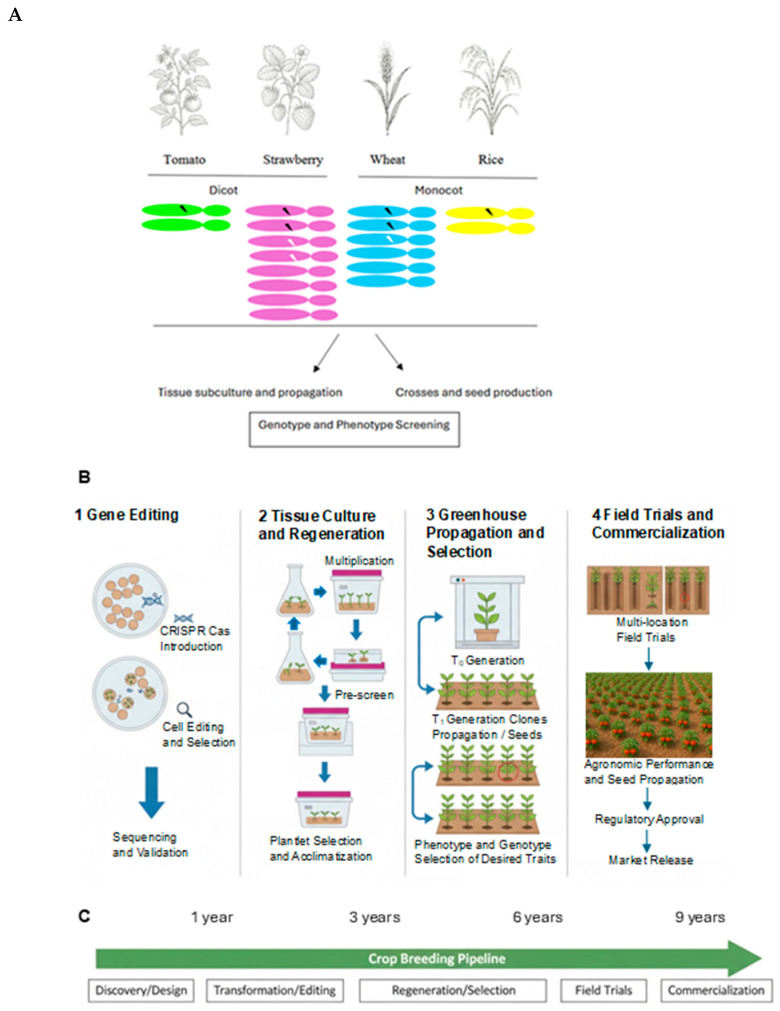
Schematic overview of key considerations and limitations associated with CRISPR–Cas-mediated crop breeding. (**A**) Gene editing of chromosomes (colored) in polyploid plants can generate dominant, codominant, incompletely dominant, additive, or recessive alleles (edits indicated by black and white zigzag arrows), with reported editing efficiencies of up to 89% in dicots and 91% in monocots [34]. (**B**) Edited tissues may be multiplied through subculture and vegetative propagation or through sexual crosses and seed production, followed by molecular screening to determine genotypic sequences, the presence of transgenic nucleic acids, and levels of heterozygosity. Selection progress is highlighted by arrows and selected line circled (red). Current CRISPR–Cas applications are largely limited to well-characterized, simply inherited traits, whereas complex quantitative trait loci remain challenging targets. (**C**) Phenotypic screening of germplasm is essential to confirm trait expression, detect deleterious off-target edits, and ensure agronomic performance. While a known single locus edit may be achieved within months for trait modification, the timeline for variety selection and release still involves many steps over several years.

CRISPR–Cas systems have transformed plant genome engineering by enabling precise, multiplexed, and increasingly transgene-free modification of agronomic traits with substantially greater speed and predictability than conventional breeding or earlier nuclease platforms. Following their adaptation for genome editing, CRISPR tools were rapidly deployed in crops to generate targeted knockouts, allele replacements, and regulatory modifications affecting yield, disease resistance, and abiotic stress tolerance (Figure 4). Compared with previous genome repair and modification, CRISPR offers simpler design, lower cost, and scalable multiplexing, features particularly valuable for editing plants recalcitrant to tissue culture propagation, redundant gene families and polyploid genomes.

As gene editing methodologies continue to evolve, their applications are expected to expand significantly in the future, especially when combined with other emerging technologies such as deep sequencing, epigenetics, and artificial intelligence [191,192,193,194,195,196,197,198,199,200,201,202,203]. The use of multiple sgRNA enables accelerated genome editing strategies for crop improvement, especially when pathways are identified that confer value-added characteristics [204]. However, technical and biological constraints remain significant; PAM requirements restrict targetable loci, efficient homology-directed repair is rare in most somatic plant tissues, delivery and regeneration are genotype-dependent and often recalcitrant, and polyploidy complicates complete allele modification. Off-target activity and regulatory heterogeneity across jurisdictions further hinder application. Thus, while CRISPR–Cas platforms provide unparalleled precision and versatility for crop improvement, their agronomic impact ultimately depends on advances in delivery systems, tissue culture independence, and deeper understanding of plant DNA repair and genome complexity.

Emerging computational and technological innovation is further expanding the scope and precision of plant genome editing. This may be especially important for crops that lack a diverse genetic gene pool and are susceptible to attack by emerging pathogens and vulnerable to extreme growing conditions [205,206,207,208,209,210,211,212,213,214,215,216,217]. The AI-assisted analysis of large genomic datasets has started to improve guide RNA design and reduce off-target activity, a persistent challenge resulting from the inherent tolerance of CRISPR systems to guide–target mismatches [5,6,7,8,9,10,11,12,13,14]. Such unintended cleavage events can generate genomic rearrangements, including deletions, inversions, or translocations, that may activate stress-response pathways, highlighting the need for improved predictive algorithms and enhanced editor specificity. AI-driven modeling and structural prediction platforms, such as SWISS-Model and AlphaFold2, also facilitate the prediction of protein-level consequences of genomic edits, thereby accelerating the identification of functional targets [218,219]. At the technological level, advances in delivery systems, including viral vectors, nanoparticles, agroinfiltration, biolistic methods, and meristematic injection, are expanding the diversity of crops amenable to genome editing while allowing greater spatial or temporal control of CRISPR activity [24,220,221,222,223].

CRISPR platforms consistently outperform earlier genome editing platforms in terms of design simplicity, multiplexing capacity, and editing efficiency, with Cas9 frequently achieving mutation rates of 50–80% in protoplasts and 10–70% in stable transformants [34,35,36,37,38,39,40,41,42,43,44,45,46,47,48,49,50,51,52,53,54,55,56,57,58,59,60,61,62,63,64,65,66,67,68,69,70,71,72,73,74,75,76,77,78,79,80,81,82,83,84,85,86,87,88,89,90,91,92,93,94,95,96,97,98,99,100,101,102,103,104,105,106,107,108,109,110,111,112,113,114,115,116,117,118,119,120,121,122,123,124,125,126,127,128,129,130,131,132,133,134,135,136,137,138,139,140,141,142,143,144,145,146,147,151,152,153,154,155,156,157,158,159,160,161,162,163,164,165,166,167,168,169,170,171,172,173,174,175,176,177,178,179,180,181,182,183,184,185,186,187,188,189,190]. As these technologies continue to evolve, they are enabling new strategies such as accelerated domestication of wild plant species, engineering of metabolic pathways for pharmaceutical or biofuel production, and optimization of crop traits through regulatory and epigenetic modification. The emergence of base and prime editing, which combine Cas9 with a reverse transcriptase and use a prime editing guide RNA (pegRNA) to specify the target site and desired edit, further extends precision by enabling predictable nucleotide substitutions without double-strand DNA breaks [224]. However, the deployment of genome-edited crops remains shaped by regulatory frameworks, intellectual property considerations, and public acceptance, all of which vary considerably among jurisdictions [19,20,225,226]. The commercialization of genome-edited tomatoes with elevated γ-aminobutyric acid (GABA) levels in Japan illustrates the growing transition of CRISPR technologies from experimental platforms to agricultural products [227], while also highlighting the complex legal and societal landscape that continues to influence their global adoption.

## 10. Conclusions

The future of gene editing in plants promises to revolutionize global agriculture and food systems. By enabling targeted modifications with high specificity and efficiency through endogenous repair mechanisms, this technology empowers researchers to address pressing issues with unprecedented speed and possibilities, from food security and population growth to environmental sustainability in response to climate and market demands. Continued interdisciplinary collaboration and responsible innovation, leveraging emerging technologies and advances, will be key to realizing the full potential of plant gene editing.

Innate RNA adaptive immunity represents an important set of tools for plant genetic research and biotechnology improving product development. CRISPR’s precision and permanence contrast with the flexibility and reversibility of RNAi-based approaches and complements existing breeding technologies. The choice of technology to address crop challenges should be guided by experimental objectives, regulatory context, and whether a transient or stable outcome is desired. For optimal results, integrated approaches leveraging various technologies will offer synergistic advantages in functional genomics and multidisciplinary crop improvement programs for industry and global populations.

## Figures and Tables

**Table 1:** Abiotic CRISPR-Cas improvements.

Trait	Plant	Locus	Reference
Drought	*Arabidopsis*	*AtOST2*	[35]
		*AtAREB1*	[36]
	*Glycine max*	*GMMYB118*	[37]
	*Orza sativa*	*OsERA1*	[38]
		*OsPUB7*	[39]
		*StDRO2*	[40]
	*Zea mays*	*ZmHDT103*	[41]
Salt	*Arabidopsis*	*AtWRKY3*, *AtWRKY4*	[42]
		*AtACQOS*	[43]
	*G. max*	*GmDrb2a*, *GmDrb2b*	[44]
		*GmAITR*	[45]
		*GmNHL1*	[46]
		*GmCG*	[47]
	*Hordeum vulgare*	*HvGSK1.1*	[48]
	*O. sativa*	*OsbHLH024*	[49]
		*OsDST*	[50]
		*OsRAV2*	[51]
		*OsRP22*	[52]
		*OsNAC45*	[53]
		*OsSPL10*	[54]
		*OsTPP3*	[55]
	*Solanum lycopersium*	*SIHyPRP1*	[56]
		*SIHAK20*	[57]
	*Solanum tuberosum*	*Stcoilin*	[58]
	*Triticum aestivum*	*TaHAG1*	[59]
	*Zea mays*	*ZmHKT2*	[60]
Heat	*Gossypium hirsutum*	*GhPGF*, *GhCLA1*	[61]
	*Lactuca sativa*	*LsNCED4*	[62]
	*O. sativa*	*GmHSP17.5Ep*	[63]
		*OsDEP1*, *OsROC5*	[64]
		*OsGER4*	[65]
		*OsHSBP1*	[66]
		*OsTMS5*	[67]
	*S. lycopersicum*	*SIAGL6*	[68]
		*SICPK28*	[69]
		*SIMAPK3*	[70]
	*T. aestivum*	*TaHsfA1*	[71]
	*Z. mays*	*ZmTMS5*	[72]
		*ZmHSPs*	[73]
Cold	*O. sativa*	*OsPRP1*	[74]
		*OsbHLH57*	[75]
	*S. lycopersicum*	*SICBF1*	[76]
	*S. tuberosum*	*StVInv*	[77]
	*T. aestivum*	*TaPGK*	[78]
	*Z. mays*	*ZmG6PDH1*	[79]
Metal	*Arabidopsis*	*Atoxp1*	[80]
	*O. sativa*	*OsARM1*	[81]
		*OsHAK1*	[82]
		*OsLCD*	[83]
		*OsLCT1*	[84]
		*OsMYB84*	[85]
		*OsNIP3*	[86]
		*OsNramp5*	[87]
		*OsNRAMP1*	[88]
		*OsZIP5*, *OsZIP9*	[89]
		*OsPRX2*	[90]
		*OsATX1*	[91]
	*T. aestivum*	*TalPK1*	[92]
Herbicide	*Brassica napus*	*BnALS*	[93]
	*Manihot esculenta*	*MeEPSPS*	[94]
	*O. sativa*	*OsALS*	[95]
		*OsTB1*	[96]
		*OsPUT1/2/3*	[97]
		*OsACC*	[98]
UV Radiation	*O. sativa*	*OsCOP1*	[99]
Oxidation	*O. sativa*	*OsCAT2*	[100]

**Table 2:** Biotic CRISPR-Cas improvements.

Biotic Stress	Pathogen/Pest	Plant	Locus	Reference
Fungi/ Fungus-like	Powdery mildew	*Cucumis sativus*	*CsMLO8*	[101]
		*Petunia* × *hybrida*	*PhMLO*	[102]
		*Solanum lycopersicum*	*SIMLO*	[103]
		*S. lycopersicum*	*SIPMR4*	[104]
		*Vitis vinifera*	*VvMOL3*	[105]
	Late blight	*S. lycopersicum*	*miR482b/c*	[106]
		*Solanum tuberosum*	*S genes*	[107]
			*CCoAOMT*	[108]
		*Solanum americanum*	*SaNRL1*	[109]
	Gray mold	*S. lycopersicum*	*SIPL*	[110]
	White mold	*Glycine max*	*Gm5g29080*	[111]
	Stripe rust	*Triticum aestivum*	*TaCIPK14*	[112]
	Southern late blight	*Zea mays*	*ZmAGO18b*	[113]
	Rice blast	*Oryza sativa*	*OsPi21*	[114]
Bacteria	Bacterial blight	*O. sativa*	*OsSWEET115*	[115]
			*OsPUB9*	[116]
		*Citrus sinensis*	*CsLOB1*	[117]
	Bacterial speck	*S. lycopersicum*	*SIJAZ2*	[118]
	Bacterial spot	*S. lycopersicum*	*SIBs5*	[119]
	Bacterial leaf streak	*O. sativa*	*OsSULTR*	[120]
	Bacterial wilt	*S. lycopersicum*	*SIPRP1/DEA1*	[121]
			*SIGAD2*	[122]
Virus	Bean yellow dwarf	*Nicotiana benthamiana*	LIR	[123]
	Beet severe curly top	*N. benthamiana*	IR	[124]
	Tomato yellow leaf curl	*N. benthamiana*	IR	[125]
	Tomato yellow leaf curl	*S. lycopersicum*	CP/Rep	[126]
	Cotton leaf curl	*N. benthamiana*	IR	[127]
	Wheat dwarf	*Hordeum vulgare*	MP/CP/IP	[128]
	Cotton leaf curl	*N. benthamiana*	Rep	[129]
	Cauliflower mosaic	*A. thaliana*	CP	[130]
	Banana streak	*Musa* spp.	ORF1, 2, 3	[131]
	Chili leaf curl	*N. benthamiana*	C + V	[132]
	Tomato yellow leaf curl	*S. lycopersicum*	*SIPelo*	[133]
	Cucumber mosaic	*N. benthamiana*	ORF1a, 3a	[134]
	Potato virus Y, S, and A	*S. tuberosum*	P3, CI, CP	[135]
	Geminiviruses	*Manihot esculenta*	IR, ORFs	[136]
	Potato spindle viroid	*S. lycopersicon*	*SIDCL2b*	[137]
	Maize rough dwarf	*Z. mays*	*ZmGDla*	[138]
	Yellow mottle	*O. sativa*	*OsCPR5.1*	[139]
Pest	Plant hopper	*O. sativa*	*OsCKX*	[140]
	Insects	*Gossypium hirsutum*	*GhMLP423*	[141]
	Chewing insects	*G. max*	*GmUGT*	[142]
	Root-knot nematode	*O. sativa*	*OsHPP04*	[143]
	Cyst nematode	*G. max*	*GmSNAP02*	[144]
	Parasitic weeds	*Sorghum bicolor*	*CCD*	[145]
Beneficial	Mycorrhizal	*Marchantia paleacea*	*CCaMK*	[146]
	Nitrogen fixation	*O. sativa*	*CYP75*	[147]

**Table 3:** Phenotypic agronomic CRISPR-Cas characteristic improvements.

Plant	Locus	Trait	Reference
*Brassica napus*	*BnITPK*	Phytic acid	[151]
*Ipomoea nil*	*DFR-B*	Flower color	[152]
*Jatropha curcas*	*JcCYP735A*, *JcCKX*	Growth	[153]
*Citrullus lanatus*	*PDS*	Color	[154]
*Glycine max*	*GmFT2a*	Flowering	[155]
*Hordeum vulgare*	*GW2.1*	Seed set	[156]
	*Hina*	Grain hardiness	[157]
	*HvHGGT*, *HvHPT*	Vitamin E	[158]
*Manihot esculenta*	*MeMSIII*	Starch synthesis	[159]
	*MeCYP79D1*	Cyanide	[160]
*Oryza sativa*	*OsIAA23*	Development	[161]
	*OsCKX*	Growth and quality	[162]
	*OsRDD1*	Photosynthesis	[163]
	*OsHHO3*	Nitrogen uptake	[164]
	*OsGn1a*, *OsGL3*	Grain number/size	[165]
	*OsBADH2*	Fragrance	[166]
	*OsPYL9*	Yield	[167]
	*OsSD1*	Lodging	[168]
	*OsRc*, *OsRd*	Red rice	[169]
*Petunia hybrid*	*PhACO*	Longevity	[170]
	*PhPDS*	Albino	[171]
*Solanum lycopersicon*	*PI*, *PG2a*, *TBG4*	Fruit shelf life	[172]
	*SIIAA9*	Parthenocarpy	[173]
*Solanum tuberosum*	*StMYB44*	Phosphate transport	[174]
	*StGBBS*	Starch quality	[175]
	*StSS6*	Starch quantity	[176]
	*StSBE*	Starch quality	[177]
	*STPDS*	Carotenoids	[178]
	*St16DOX*	Glycoalkaloids	[179]
	*StSSR2*	Glycoalkaloids	[180]
	*StPPO2*	Enzymatic browning	[181]
	*FtsZ1*	Starch granule size	[182]
*Triticum aestivum*	*TaARE1*	Nitrogen use	[183]
	*TaRPK1*	Yield	[184]
		Zn and Fe uptake	[185]
	*TaGW2*	Seed size and weight	[186]
*Zea mays*	*SSU-crt1*, *ZmPSY*	Carotenoid increase	[187]
	*ipdC*	Improved growth	[188]
	*ZmSWEET1b*	Sugar transport	[189]
	*Zmbadh2*	Aroma	[190]

## Data Availability

No new data were created or analyzed in this study. Data sharing is not applicable to this article.
